# Supplementary material for: WordCommentsAnalyzer: A windows software tool for qualitative research
Source: F1000Res. 2018 Sep 4;7:536. Originally published 2018 May 3. [Version 2] doi: 10.12688/f1000research.14819.2 (PMC6137411; doi:10.12688/f1000research.14819.2)
Supplement: Supplementary file 3 [file f1000research-7-17614-s0002.tgz › b2a79496-4ada-47c4-9682-a99ce867836d.zip!/Eight Iranian Health Journals/Journal of Knowledge and Health_40.docx]

© Journal of Knowledge and Health. This work is licensed under CC BY 4.0 (https://creativecommons.org/licenses/by/4.0/).

Volume & Issue: دوره 3، شماره 1: 1387

Title: بررسی تأثیر استفاده از بيماران مجازي در آموزش جراحی دانشجويان پزشکي: يک مطالعه مداخله¬ای تصادفي کنترل شده

http://knh.shmu.ac.ir/index.php/site/article/view/197

مقدمه: هدف اين مطالعه بررسي تأثیر استفاده از بيماران مجازي در آموزش درد شکم در بیمار جراحي، به دانشجويان پزشکي در ابعاد مختلف مهارت­هاي باليني به­خصوص در حل مسايل باليني مي­باشد. مواد و روش­ها: در اين مطالعه مداخله­اي تعداد 48 دانشجوي دوره­ي کارآموزي بخش جراحي به دو گروه مداخله و کنترل به­صورت تصادفي تقسيم شده و در گروه مداخله، آموزش در طي 7 جلسه و با استفاده از 14بيمار شبيه­سازي شده در رابطه با دردهاي شکمي انجام شده است. در پايان مطالعه، هر دو گروه با استفاده از آزمون کتبي ساختار يافته­ي 7 قسمتي مورد ارزيابي قرار گرفتند. نتايج: در اين مطالعه از 48 دانشجوي پزشکي شرکت­کننده، تعداد 45 نفر با آزمون کتبي مورد ارزيابي قرار گرفتند. نتايج نشان مي­دهد که روش معرفي شده در افزايش مهارت­ گرفتن شرح حال به­طور معناداري مؤثر بوده در حالي­که در زمينه­ي استفاده از تست­هاي آزمايشگاهي و عکس­هاي راديولوژي، تشخيص افتراقي و درمان مناسب و هم­چنين در ارتقاء دانش آن­ها مؤثر نبوده است. در گروه مداخله، آزمون مبتني بر رايانه با استفاده از بيمار مجازي انجام شده که نتايج آن با آزمون کتبي هم­بستگي پاييني را نشان مي­دهد. نتيجه­گيري: آموزش با استفاده از بيماران مجازي در افزايش مهارت گرفتن شرح حال از بيماران مؤثر بوده در حالي­که بر ساير جنبه­هاي مهارت­هاي باليني دانشجويان تأثير معناداري نداشته است.

Title: بررسي احتمال بقای يک¬ساله و عوامل مؤثر بر آن در بيماران سکته قلبي

http://knh.shmu.ac.ir/index.php/site/article/view/198

مقدمه: بيماري­هاي قلبي عروقي مهم­ترين عامل مرگ­ومير در اغلب کشورهاي جهان شناخته شده­اند و طبق آمارهاي رسمي، ميزان مرگ­ومير ناشي از اين پديده در ايران رو به افزايش است. با توجه به نقش متغيرهاي مختلف در روند بيماري، در اين تحقيق تلاش شده مقدار احتمال بقای و ارتباط آن با متغيرهاي مختلف مؤثر در ميزان بقای بیماران مبتلا به سکته قلبی مورد مطالعه قرار گيرد. مواد و روش­ها: مطالعه انجام شده يک مطالعه هم­گروهي تاريخي مي­باشد که 113 بيمار بستري در بخش CCU بيمارستان امام حسين (ع) شاهرود با تشخيص سکته حاد قلبي در شش ماه دوم سال 1382 و 1383 را مورد بررسي قرار داده است. در اين مطالعه ضمن بررسي سرانجام بيماري تا يک­سال پس از سکته قلبي، اطلاعاتي در مورد سن، جنس، مصرف سيگار، سابقه ديابت، دريافت و عدم دريافت استرپتوکيناز نيز استخراج گرديد و اثر آن بر سرانجام بیماری (Outcome) با استفاده از روش حاصل­ضرب کاپلان- ماير بررسي شد. داده­ها با استفاده از نرم افزار SPSS و Epi-Info آناليز گرديد. نتايج: ميانگين سن بيماران 1/13±9/63 سال بود. از اين افراد تحت مطالعه، 6/64 درصد مرد و 9/23 درصد سيگاري بوده­اند. 1/53 درصد سابقه فشار خون بالا، 8/24 درصد سابقه ديابت، 4/35 درصد سابقه بيماري­هاي ايسکميک قلبي و 2/6 درصد نيز بلوک قلبي داشته­اند. در طي يک­سال 36 نفر (9/36%) از بيماران بر اثر بيماري قلبي فوت نموده­اند. احتمال بقای يک­ساله در مردان 9/69% و در زنان 0/65% بوده که تفاوت معناداري را نشان نمي­دهد. احتمال بقای يک­ساله در بيماران دريافت­کننده استرپتوکيناز (6/81%) به­طور معناداري بالاتر از بيماراني بود که استرپتوکيناز دريافت نکرده بودند اين اختلاف پس از حذف اثر سيگار نيز معنادار بود (05/0>P). نتيجه­گيري: در اين مطالعه مصرف استرپتوکيناز به­عنوان يک داروي ترومبوليتيک در ميزان بقای بيماران مؤثر بوده در حالي­که جنسيت و سابقه بيماري­هاي ايسکميک قبلي و ديابت در اين بيماران بر احتمال بقای يک­ساله تأثير ندارند.

Title: بررسی مرگ¬ومیر کودکان زیر یک¬سال و عوامل مؤثر بر آن در شهر بیرجند

http://knh.shmu.ac.ir/index.php/site/article/view/199

مقدمه: اولين سال زندگي براي تأمين زير بناي سلامت و بهبود كيفيت زندگي اهميت ويژه­اي دارد. در سال­های اخیر میزان مرگ­ومیر کودکان زیر یک­سال در کشورهای پیشرفته کاهش یافته اما هم­چنان در کشورهای در حال توسعه بالا می­باشد و شناخت عوامل مرتبط با این مرگ­ها می­تواند قدم مهمی برای کاهش این شاخص باشد و این مطالعه با هدف بررسی فاکتورهای مرتبط با این مرگ­ها در شهرستان بیرجند طراحی گردید. مواد و روش­ها: دراین مطالعه مورد شاهدی تمام مرگ­های کودکان زیر یک­سال اتفاق افتاده در بیمارستان­های شهر بیرجند در مدت یک­سال به­عنوان گروه مورد در نظر گرفته شدند. برای هر مورد، دو شاهد انتخاب گردید. انتخاب شاهدها براساس نزدیک­ترین فاصله زمانی از نظر تولد به مورد بوده که تا یک­سالگی زنده مانده­اند و محل سکونت آن­ها همان منطقه موردها بود که در شهر از دفتر آمار حیاتی مراکز بهداشتی- درمانی شهری و در روستا از خانه­های بهداشت روستایی استخراج گردیدند. اطلاعات از طريق پرسش­نامه با مصاحبه با مادر و بررسی پرونده فوت كودك جمع­آوري و تجزيه و تحليل گرديد. نتايج: در اين مطالعه 7/79% از موارد مرگ كودكان زير يك­سال در ماه اول زندگي و 3/42% بين يك­ماه تا يك­سال اتفاق افتاده بود. مهم­ترين علت فوت در نوزادان نارسي و عارضه كمبود وزن هنگام تولد (2/54%) و مهم­ترين علت مرگ در شيرخواران يك­ماه تا يك­سال ناهنجاری مادرزادی (3/33%) تعيين گرديد. در بررسي فاكتورهاي مؤثر بر مرگ­ومير شيرخواران كه بر اساس آناليز رگرسيون لجستيك شرطي صورت گرفت، متغيرهاي سواد پدر، فاصله تولد با كودك قبلي، وضعيت نوزاد هنگام تولد، وزن نوزاد هنگام تولد، نوع زایمان، بارداری پرخطر ارتباط معني­داري با مرگ­ومير شيرخواران نشان داد. نتیجه­گیری: توجه ويژه به دوران نوزادي و مراقبت ويژه از نوزاداني كه از مادران در معرض خطر متولد مي­شوند، و ارایه آموزش­هاي بهداشتي و مراقبت ويژه از اين مادران مي­تواند نقش مؤثري در كاهش مرگ­ومير شيرخواران داشته باشد.

Title: بررسی عوامل غذایی دریافتی مرتبط با کم‌خونی فقر آهن در بین زنان غیرباردار 15 تا 45 ساله زنجان

http://knh.shmu.ac.ir/index.php/site/article/view/200

مقدمه: کم­خونی تغذيه­ای يکی از مشکلات بهداشت عمومی در سرتاسر جهان مخصوصاً در کشورهای درحال توسعه می­باشد. زنان به­ويژه در سنين باروری جزو گروه­های در معرض خطر می­باشند. حدود یک سوم جمعيت جهان ازکم­خونی رنج می­برند که کمبودهای تغذيه­ای مهم­ترين علت اين کم­خونی­ها به­شمار می­آيد. حدود 350 ميليون زن و 50 درصد کودکان در کشورهای در حال توسعه کم­خون هستند. اين بيماری در کشور ما نيز شيوع نسبتاً بالايی داشته و طبق آمار ارایه شده توسط دفتر بهبود تغذيه جامعه 50 درصد زنان 15 تا 49 ساله کشور به درجات مختلف کم­خونی مبتلا بوده و 34 درصد آنان کمبود شديد آهن دارند. لذا با توجه به اين تاکنون هيچ­گونه بررسی در اين مورد در زنجان انجام نشده بود، اين بررسی با هدف تعيين ميزان شيوع کم­خونی در بين زنان 15 تا 45 ساله غيرباردار شهر زنجان و ارتباط آن با انرژی و برخی مواد مغذی دريافتی طراحی و اجرا گرديد. مواد و روش­ها: مطالعه از نوع مقطعی بود که تعداد 33 خوشه با استفاده از نقشه شهر و روش تصادفی انتخاب و از هر خوشه 10 نفر جمعاً 330 نفر زنان 15 تا 45 ساله مورد مطالعه قرار گرفتند. اطلاعات با استفاده از پرسش­نامه عمومی، پرسش­نامه 24 ساعت يادآمد خوراک و آزمايش نمونه­های خون جمع­آوری گرديد. آزمايشات هموگلوبين، هماتوکريت، MCHC، آهن سرم، TIBC، اشباع ترانسفرين و فريتين انجام گرديد و سپس ميانگين آن­ها تعيين و با استفاده از معيارهای تعيين شده ميزان شيوع کم­خونی ناشی از کمبود آهن براساس فراسنج­های خونی اندازه­گيری شد و سپس با استفاده از آزمون 2χ، وابستگی بين فراسنج­ها با انرژی، پروتئين، آهن و ويتامين C تعيين گرديد. نتایج: شيوع کم­خونی ناشی از فقرآهن براساس هماتوکریت 3/47% وMCHC  5/14% به­دست آمد که هم­بستگی معنی­داری بين کليه فراسنج­ها با انرژی، پروتئين و ويتامين C دريافتی به­جز هماتوکريت با پروتئين به­دست آمد. در این مطالعه 5/11 درصد کم­تر از مقادیر توصیه شده RDA دریافت انرژی داشتند. هم­چنین 4/15 درصد کمبود دریافت پروتیئن و 5/31 درصد آنان کمبود دریافت ویتامین C بر اساس مقادیر توصیه شده داشتند. نتیجه­گیری: شيوع کم­خونی ناشی از فقرآهن در زنان مورد بررسی در حد متوسط به بالا بود با توجه به نقش پروتئین­ها و ویتامین C در افزایش جذب آهن، آموزش تغذيه و تجويز مکمل آهن به اين گروه توصيه می­گردد.

Title: علاقه¬مندي به تحصيل در رشته مامایي و عوامل مرتبط با آن در دانشجویان دانشگاه¬های علوم پزشکی تهران

http://knh.shmu.ac.ir/index.php/site/article/view/201

مقدمه: از ابتدايي­ترين لوازم و مقتضيات يادگيري علاقه و رغبت به آموزش است. در واقع علاقه مركب از انگيزش و كنجكاوي است و فرد برانگيخته براي دستيابي به آگاهي­هاي بيش­تر وكسب موفقيت پافشاري و ممارست بيش­تري خواهد كرد. لذا علاقه­مندي فراگيران نسبت به رشته تحصيلي­شان از عوامل مهم پيشرفت برنامه­هاي آموزشي و نهايتاً بازدهي و مسئوليت­پذيري آتي آنان مي­باشد و بايد همواره به آن توجه كافي مبذول گردد. اين مطالعه به­منظور تعيين ميزان علاقه­مندي دانشجويان مامايي دانشگاه­هاي علوم پزشكي شهر تهران نسبت به رشته تحصيلي­شان در سال 1385 صورت گرفت. مواد و روش­ها: در اين پژوهش توصیفی از روش نمونه­گيري سرشماری استفاده شد و کلیه 260 دانشجوی کارشناسی پیوسته مامایی پذیرفته شده در یکی از 3 دانشگاه علوم پزشکی شهر تهران كه در ترم­هاي اول تا هشتم مشغول به تحصيل بودند وارد مطالعه شدند. ابزارگردآوری داده­ها پرسش­نامه بود، كه در دو بخش مشخصات دموگرافیک و عوامل مرتبط با سنجش ميزان علاقه­مندي طراحی گردید. جهت تعیین اعتبار ابزار از روش اعتبار محتوی و برای پایایی آن از روش آزمون مجدد استفاده شـد. داده­ها با استفاده از آزمون­هاي آماري كاي اسكوار و ضريب هم­بستگي اسپيرمن و نرم­افزار SPSS مورد تجزيه و تحليل قرار گرفت. نتایج: یافته نشان داد كه ميزان علاقه­مندي دانشجويان نسبت به رشته تحصيلي­شان پايين با ميانگين امتياز 5/37% بود و اغلب آنان (8/80%) با علاقه اقدام به انتخاب رشته نكرده­اند. مهم­ترين عوامل مرتبط با گرايش دانشجويان نسبت به تحصيل در اين رشته، مفيد واقع شدن آن در تمام مراحل زندگي فردي و وجود تجربيات مهيج در حرفه مامايي بود. بين تحصيلات و شغل والدين، ترم تحصيلي و ميزان علاقه­مندي دانشجويان نسبت به رشته تحصيلي­شان ارتباطي ديده نشد. نتيجه­گيري: با توجه به اينكه فراگيران از علاقه كافي براي تحصيل در اين رشته برخوردار نبودند و تنها ماهيت شغلي اين حرفه مورد توجه دانشجويان بود مي­توان اين­گونه قضاوت كرد كه ايشان حرفه آينده خود را چندان مطابق با نيازها و انتظارات­شان ارزيابي نكرده و بديهي است كه عواقب سوء ناشي از اين مسئله متوجه نظام آموزش مامايي و سيستم سلامت كشور خواهد بود. لذا متصديان اين گروه آموزشي بايد با شناخت معضلاتي كه منجر به عدم رغبت دانشجويان نسبت به رشته تحصيلي­شان مي­شود و با ارایه راه­كارهاي مؤثر براي حل آن­ها موجبات ارتقاء آموزش، علاقه­مندي و بازدهي آن­ها را فراهم آورند. هم­چنين لزوم تجديد نظر در نحوه جذب دانشجو مطابق با نيازهاي فردي و اجتماعي و در جهت حفظ منزلت افراد و مشاغل جهت جلوگيري از به هدر رفتن سرمايه­هاي مادي و معنوي فرد و جامعه اساسي است.

Title: ارزيابي و مقايسه تشخيص¬هاي كلينيكي و پاراكلينيكي واژنيت تريكومونایي در زنان مراجعه¬كننده به مراكز بهداشتي درماني شهرستان شاهرود

http://knh.shmu.ac.ir/index.php/site/article/view/202

مقدمه: تريكومونياز يكي از بيماري­هاي منتقل شونده از راه جنسي است كه عامل آن يك انگل فلاژل­دار به­نام تريكوموناس واژيناليس مي­باشد و سومين علت شايع واژينيت است. اين بيماري 180 ميليون زن را در تمام جهان و 2 تا 3 ميليون زن آمريكايي را در هر سال مبتلا مي­سازد. با توجه به شيوع بالاي تريكومونا و هزينه­هاي سرسام آور درماني بر آن شديم تا تشخيص­هاي كلينيكي و پاراكلينيكي را مورد ارزيابي و مقايسه قرار دهيم. مواد و روش­ها: پژوهش حاضر يك مطالعه توصيفي ـ تحليلي است كه بر روي 300 خانم مراجعه­کننده با علایم عفونت­هاي واژينال انجام شده است. هم­زمان با معاينه فيزيكي با اسپي كولوم دو نمونه گسترش ترشحات ابتدا از آندوسرويكس و سپس اگزوسرويكس بر روي لام تهيه شد. سپس يك نمونه از ترشحات كلدوساك خلفي بيمار همراه با Cotton Soup گرفته و داخل لوله آزمايش حاوي سرم فيزيولوژي استريل قرار داده شد و پرسش­نامه اول توسط كارشناس مامائي تكميل شد و انجام آزمايشات پاپ اسمير، اسمير مرطوب و كشت و تكميل پرسش­نامه دوم توسط كارشناس آزمايشگاه انجام گرديد. در انتها نتايج تشخيص­هاي باليني، ميكروب شناسي و سيتولوژي با هم مقايسه گرديد و از طريق روش­هاي آماري شاخص­هاي حساسيت روش­هاي مختلف تشخيص مورد بررسي قرار گرفت. نتایج: نتايج پژوهش نشان دهنده آن بود كه بيش­ترين سن شيوع واژنيت تريكومونایي با تشخيص باليني 21 تا 35 سال (6/52%) و كم­ترين آن گروه سني 20 سال و كم­تر (3/10%) بوده است. بر­اساس نتايج باليني 78 مورد تريكومونا گزارش شده است در حالي­كه در هيچ مورد از محيط­هاي كشت تريكومونا ثابت نشده است، نتايج نشان مي­دهد كه روش تشخيص باليني نسبت به كشت باعث 26% مثبت كاذب در تشخيص تريكومونا بوده و در محيط اسمير مرطوب و پاپ اسمير 1 مورد از تريكومونا ثابت شده است. نتايج نشان مي­دهد كه روش تشخيص باليني نسبت به اسمير مرطوب و پاپ اسمير 7/25% مثبت كاذب در تشخيص تريكومونا داشته است. نتيجه­گيري: استفاده از پاراكلينيك هم­زمان با معاينات كلينيكي نقش مهمي را در تشخيص و درمان واژنيت­ها ايفا مي­كند و لذا به­منظور اجتناب از تجويز غيرضروري دارو و پيش­گيري از عوارض ناشي از مصرف داروها انجام برخي آزمايشات پاراكلينيكي در بيماران مشكوك توصيه مي­گردد.

Title: تأثیر ویتامین Cتکمیلی بر سطح قند و چربی خون در بیماران دیابتی نوع II

http://knh.shmu.ac.ir/index.php/site/article/view/203

مقدمه: افراد ديابتي بيش­تر از افراد عادي در معرض عوارض متعدد قلبي- عروقی، عصبی، کلیوی، چشمی می­باشند. از آن­جا­که ويتامين C در بسياري از فعل و انفعالات شيميايي به­طور رقابتي جانشين گلوکز مي­شود و از گليکوزيله شدن غير آنزيمي پروتئين­ها از جمله هموگلوبين و لیپوپروتئین­ها جلوگيري مي­کند، به نظر مي­رسد در پیش­گیری از عوارض دیابت مؤثر باشد. در این مطالعه تأثیر ویتامین C تکمیلی بر روی میزان قند و چربی بیماران دیابت نوع II مورد بررسی قرار گرفته است. مواد و روش­ها: اين مطالعه از نوع کارآزمايي باليني تصادفی دو سوکور می­باشدکه بر روي 136 بيمار مبتلا به ديابت نوع II انجام شده است. بیماران به­طور تصادفی در دو گروه دریافت­کننده ویتامین C (به مقدار 1250 گرم، پنج بار در روز، به مدت 3 ماه) و دارونما قرار گرفتند، میزان قند خون ناشتا، هموگلوبین گلیکوزیله، کلسترول، تری­گلیسرید LDL ,HDL آن­ها  قبل و بعد ازمطالعه اندازه­گیری شد و اطلاعات به­دست آمده با استفاده از آزمون t نمونه­هاي زوج و نرم­افزار SPSS مورد تجزیه و تحلیل قرار گرفت. نتايج: در این مطالعه اکثریت بیماران را زنان تشکیل داده­اند (در گروه دريافت­كننده ويتامين C، 7/64% و گروه شاهد 2/62%). ميانگين سني گروه دريافت­كننده ويتامين C برابر 8/6 ± 8/51 سال و گروه دارونما (شاهد) 7/9 ± 8/50 سال گزارش شده است. در پایان مطالعه در گروه مصرف­کننده ویتامین C نسبت به گروه شاهد ميانگین غلظت FBS، LDL، تري­گليسريد، كلسترول و هموگلوبين گليكوزيله به­طور معنا­داری کاهش و ميانگين HDL در گروه مصرف­کننده ویتامین C افزایش معنا­داری داشته است. نتیجه­گیری:  نتایج مطالعه نشان می­دهد که ویتامین C تکمیلی، میزان قند و چربی خون را در بیماران دیابتی نوع دو کاهش می­دهد. لذا مصرف آن را می­توان به بیماران دیابتی توصیه نمود و به­این وسیله باعث کاهش عوارض این بیماری خواهیم بود.

Title: بررسی افسردگی و سلامت عمومی در بیماران دیابتی نوع II

http://knh.shmu.ac.ir/index.php/site/article/view/204

مقدمه: افسردگی اختلال پر عارضه و ناتوان کننده­ای است که در اغلب مطالعات شیوع آن در بیماران دیابتی بیش­تر از جمعیت عمومی است ولی رابطه­ی علیتی آن با دیابت مشخص نیست اما این اختلال سبب کاهش میل، انگیزه و احساس توانایی افراد جهت پرداختن به فعالیت­های خود مراقبتی می­گردد. این مطالعه با هدف بررسی افسردگی و سلامت عمومی بیماران دیابتی نوع II در شاهرود انجام شده است. مواد و روش­ها: در این مطالعه مقطعی 100 بیمار دیابتی مراجعه­کننده به مرکز دیابت شهرستان شاهرود مورد بررسی قرار گرفتند. داده­های مطالعه با استفاده از چک لیست داده­های دموگرافیک و پرسش­نامه افسردگی بک و سلامت عمومی (GHQ) جمع­آوری و آنالیز شده است. نتایج: شیوع افسردگی بر اساس تست بک در افراد دیابتی مورد مطالعه 78% بود و اکثریت آن­ها (32 نفر) دارای نمره بین 10 تا 18 (افسردگی خفیف) بودند. در این مطالعه بین شدت افسردگی و سن رابطه معناداری مشاهده شد (01/0=P). هم­چنین بررسی ابعاد مختلف سلامت عمومی بیماران نشان داد که بین ابعاد سلامت عمومی و شدت افسردگی رابطه معناداری وجود دارد. بر اساس نمره آزمون سلامت عمومی، 62% بیماران از نظر روان­شناختی با مشکل مواجه بودند. نتیجه­گیری: نتایج مطالعه نشان می­دهد که فراوانی علایم افسردگی در بیماران دیابتی بالا می­باشد، با توجه به غربال­گری آسان علایم افسردگی در بیماران، شناسایی بیماران افسرده و درمان آن­ها ضروری به­نظر می­رسد.
